# Supplementary material for: Expression of NAC1 Restrains the Memory Formation of CD8+ T Cells during Viral Infection
Source: Viruses. 2022 Aug 4;14(8):1713. doi: 10.3390/v14081713 (PMC9414488; doi:10.3390/v14081713)
Supplement: Supplementary file 1 [file viruses-14-01713-s001.zip › viruses-1852956-supplementary.pdf]

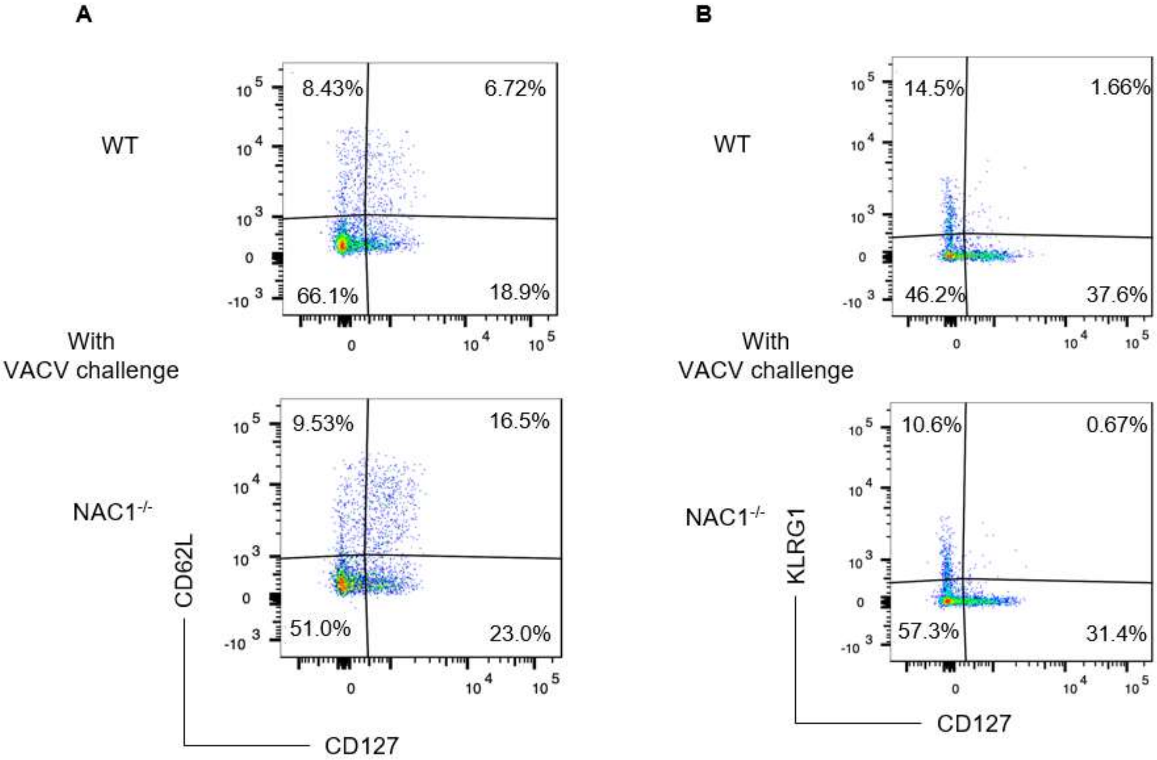

Figure S1. NAC1 alters the differentiation of memory precursors. WT or NAC1$^{-/-}$ mice were challenged with VACV at 2X10^{6} PFU/mouse. On day 7, the spleen and LNs (superficial cervical, axillary, brachial, and inguinal nodes) were dissected, smashed, and stained with CD8, CD127, CD62L, KLRG1 Abs and B8R tetramer. (**A**) After CD8^{+} B8R^{+} gating, the CD8^{+} B8R^{+} CD127^{+} CD62L^{+} and CD8^{+} B8R^{+} CD127^{+} CD62L^{-} populations were analyzed by flow cytometry. (**B**) After CD8^{+} B8R^{+} gating, the CD8^{+} B8R^{+} CD127^{-} KLRG1^{+} population was analyzed by flow cytometry. The plotting data shown are representative of three identical experiments (n=5).

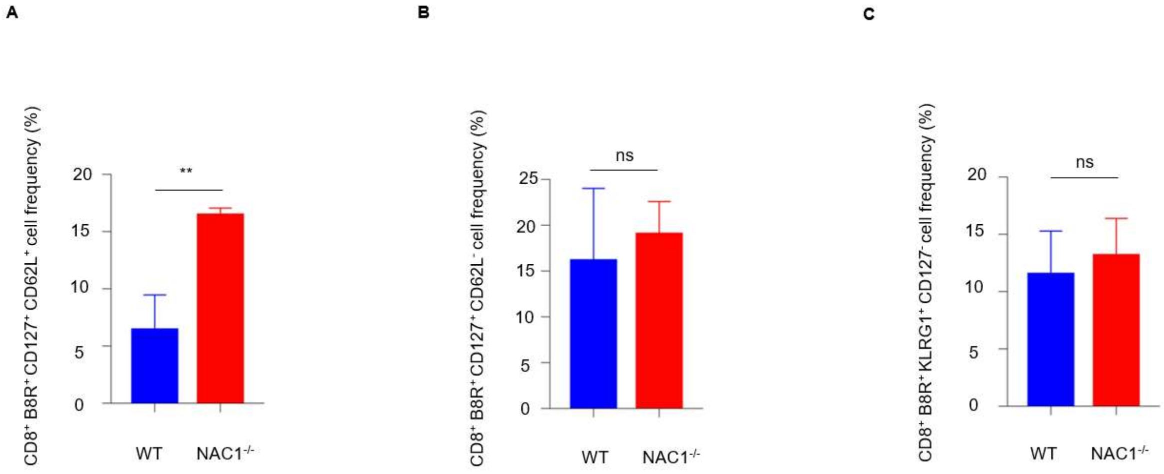

Figure S2. Enhanced CD8^{+} B8R^{+} CD127^{+} CD62L^{+} was observed in NAC1$^{-/-}$ mice. Quantification for the different populations was performed on day 7 after the VACV challenge. (**A**) CD8^{+} B8R^{+} CD127^{+} CD62L^{+} cell frequency. P-value is 0.004. (**B**) CD8^{+} B8R^{+} CD127^{+} CD62L^{-} cell frequency. The *p*-value is 0.583. (C) CD8^{+} B8R^{+} CD127^{-} KLRG1^{+} cell frequency. P-value is 0.586. (**, $p < 0.01$; ns, $p > 0.05$).

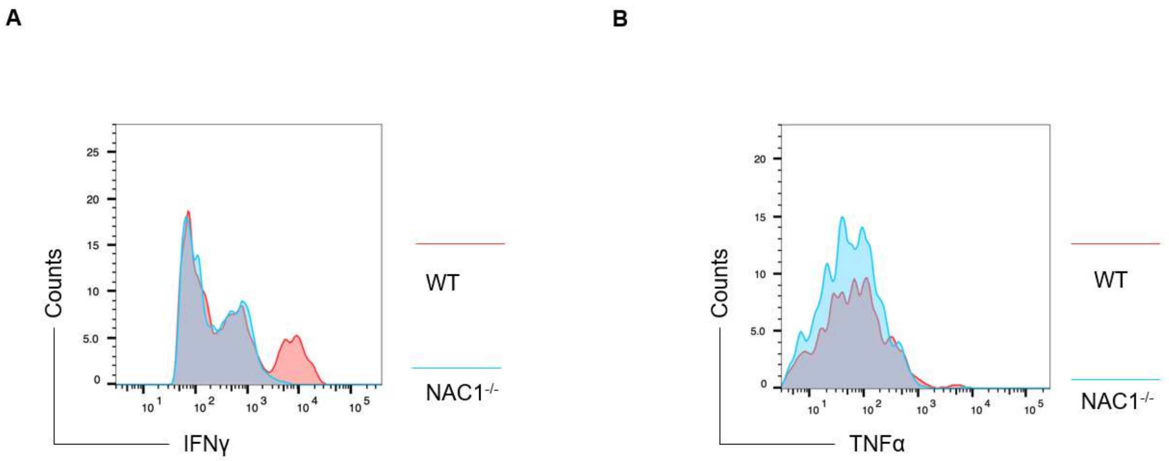

Figure S3. Functional cytokines are influenced by NAC1 in VACV-specific CD8$^+$ T cells. WT or NAC1$^{-/-}$ mice were challenged with VACV at 2X10^6 PFU/mouse. On day 7, the spleen and LNs (superficial cervical, axillary, brachial, and inguinal nodes) were dissected, smashed, and stained with CD8, IFNγ, TNFα Abs and B8R tetramer. (**A**) IFNγ production was analyzed by flow cytometry after CD8$^+$ B8R$^+$ gating. (**B**) TNFα production was also analyzed by flow cytometry after CD8$^+$ B8R$^+$ gating.
